# Fall classification, incidence and circumstances in patients undergoing total knee replacement

**DOI:** 10.1038/s41598-022-23258-x

**Published:** 2022-11-18

**Authors:** José-María Blasco, José Pérez-Maletzki, Beatriz Díaz-Díaz, Antonio Silvestre-Muñoz, Ignacio Martínez-Garrido, Sergio Roig-Casasús

**Affiliations:** 1 Group in Physiotherapy of the Ageing Process: Social and Healthcare Strategies, Departament de Fisioteràpia, Universitat de Valéncia (Spain), Calle Gascó Oliag nº5, 46010 Valencia, Valencia Spain; 2 IRIMED Joint Research Unit, IIS La Fe - UV, Valencia, Spain; 3 Hospital Clínic i Universitari de València, Valencia, Spain; 4 Hospital Universitari i Politècnic La Fe, Valencia, Spain

**Keywords:** Health care, Rheumatology, Orthopaedics

## Abstract

The objective was to propose a fall-classification framework for patients undergoing total knee replacement (TKR). In addition, we reinforced the available evidence on fall incidence and circumstances and compared the characteristics of fallers versus. nonfallers. Retrospective and prospective data were collected from 253 subjects with severe knee osteoarthritis who were waiting for primary TKR. Falls were classified considering the location of the destabilizing force, source of destabilization and fall precipitating factor. Fall incidence and circumstances were described; the characteristics of fallers and nonfallers in terms of functional and balance performance were compared with *F*-tests (95% CI). The fall incidence before surgery was 40.3% (95% CI 34.2% to 46.6%). This figure decreased to 13.1% (95% CI 9.2% to 18.0%) and to 23.4% (95% CI 17.8% to 29.6%) at 6 and 12 months after surgery, respectively. Most falls were caused by destabilizations in the base of support (n = 102, 72%) and were due to extrinsic factors (n = 78, 76%) and trip patterns. Significant differences between fallers and nonfallers were found in knee extensor strength and monopodal stability in the surgical limb (*p* < 0.05). Falls are prevalent in patients with severe knee osteoarthritis. Symptoms and functional performance improve after surgery, and fall incidence is reduced. Most fall events originate from disruptions in the base of support and are precipitated by extrinsic factors, generally trips during walking activities.

## Introduction

Persistent pain, limited functionality, joint stiffness, quadriceps weakness and impaired proprioception are symptoms commonly reported in individuals with advanced to severe knee osteoarthritis^1–5^. These symptoms have been identified as potential contributors to knee instability, altered gait patterns and balance deficits, which increase the risk of falls^6–8^.

Precisely, fall prevention is one of the main challenges of modern societies due to its economic and socio-sanitary burden. The impact of falls will become more evident in the coming years, derived from the increase in life expectancy^9^. Currently, approximately one-third of people over 65 fall once a year^10^. Age-matched individuals with knee OA are at higher risk since the reported fall prevalence is between 23 and 63%^8^. In the end-stages of the condition, the solution of choice is known as total knee replacement (TKR), a cost-effective procedure that alleviates the effects of potential fall contributors. It also decreases fall prevalence figures, reported to be between 13 and 42% at six months to one year after surgery^8,11^. However, a high proportion of patients present residual deficits, and preoperative risk factors are not always resolved^12^. Therefore, falls are an important concern in patients undergoing TKR.

Overall, evidence-based fall-risk assessment is used to classify individuals into fallers and nonfallers, relying on three main criteria:^13,14^ previous history of falls, fall and fall-risk prediction, and clinical assessment. Advances in technology have allowed the incorporation of sensor-based systems to provide standardized clinical data to help the design of tailored interventions^15^. The available studies on patients with TKR have focused on designing balance-oriented interventions;^9,16–18^ describing patients’ clinical and demographic characteristics^19,20^; identifying risk factors;^21–23^ developing and validating clinical tests to assess fall risk;^24–26^ and characterizing the biomechanics of postural control strategies to recognize potential fallers^27,28^. Some studies have also paid attention to characterizing patients and circumstances surrounding fall events. This allowed us to classify falls according to some dominant circumstances to obtain homogenous groups that allow us to describe risk factors in certain types of falls, identifying intrinsic differences between fallers and no fallers or the type of fall, such as gender or age, or grouping factors precipitating falls that may be intrinsic or extrinsic to the individual^20–22,29,30^ However, no universal fall classification framework similar to that proposed in other populations, such as older adults or lower limb prosthesis users, ^31,32^ has been developed to date. This information may help to elucidate whether falls are due to internal or external factors, patients are more susceptible to trips, slips or some other failures, or the task(s) performed at the time of the fall^31^. It can also guide the design of oriented interventions for potential fallers and set the needs for future research.

Based on previous literature and considering the characteristics of the population under study, the main objective was to propose a framework to classify falls among patients undergoing TKR. Other objectives were to reinforce the available evidence on fall incidence and circumstances and to characterize and compare fallers vs. nonfallers. The hypothesis was that the proposed fall classification would be suitable for the population under study.

## Methods

### Design and participants

This was a cohort study including retrospective and prospective data. The design adhered to the ethical recommendations set in Helsinki and successive updates and was approved by the ethics committees of Hospital Hospital Universitari I Politècnic La Fe and Hospital Clínic Universitari from Valencia (no. 2018/0621 and no. 2018/280). Patients were recruited from such institutions, and basal assessments were conducted from March to May 2019. The Universitat de València was responsible for the integrity and conduct of the study. Potential participants were referred by two orthopedic surgeons to check compliance with eligibility criteria.

### Study subjects

**Figure 1:**
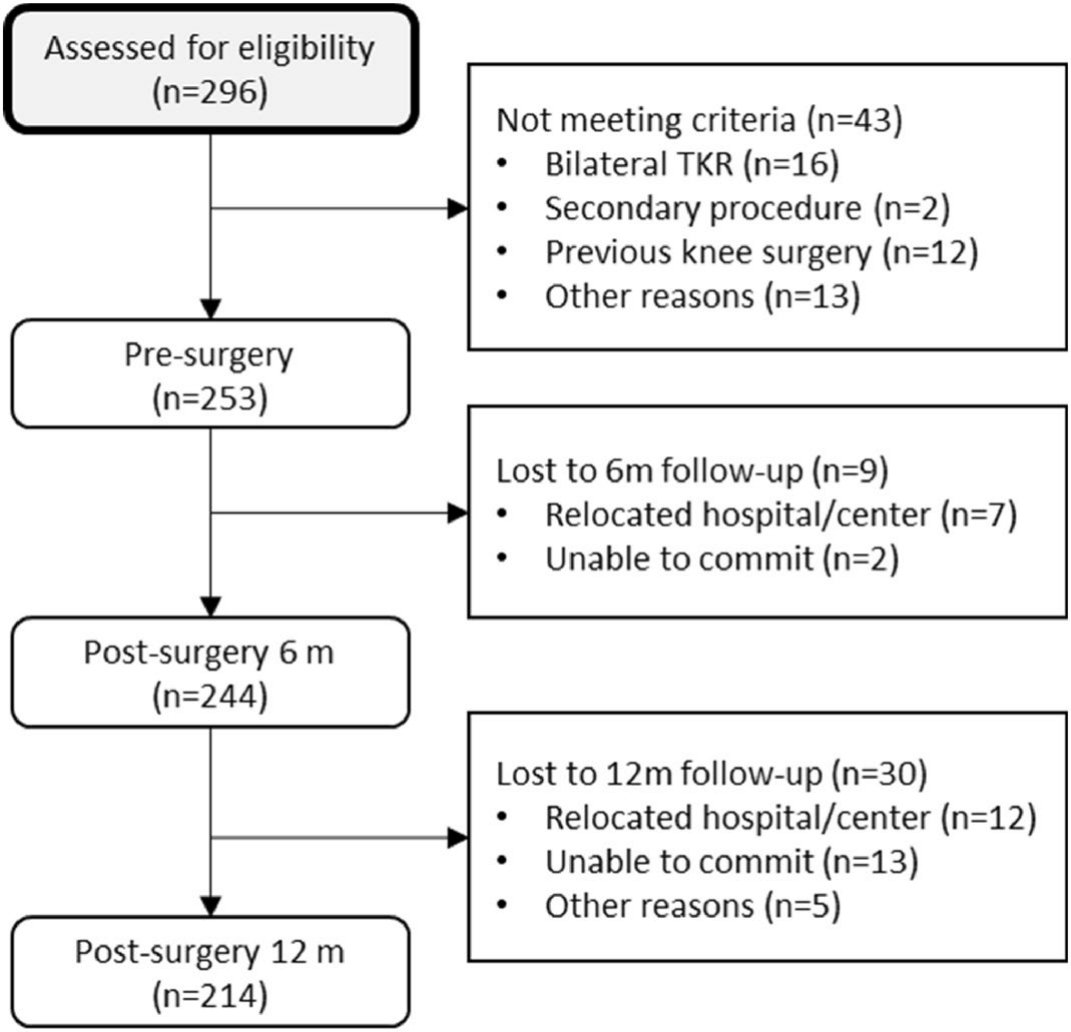
Flowchart of participants.

Subjects with severe knee osteoarthritis (Kellgren–Lawrence scale ≥ 4) on the waiting list for primary total knee replacement were eligible. Subjects were excluded if they had undergone previous nonarthroplasty surgery (i.e. arthroscopy, osteotomy) or presented with diagnosed osteoarthritis in any other joint of the lower limbs or other significant musculoskeletal or neurological condition. Of the 296 potential participants, 253 were eligible and agreed to participate. The main reasons for exclusion were bilateral and/or secondary surgical procedure, previous knee surgery, or diagnosed condition (e.g., vestibular affection, OA in other limb). All participants were verbally informed about the study and signed an informed consent form to participate. The flowchart of participants throughout the study is shown in Fig. 1.

### Procedures

The characteristics of participants were collected in face-to-face interviews, including sex; age; weight; operated knee; residence (rural, city); and use of walking aids. Participants were assessed before (baseline) and after surgery (at 6 and 12 months) by two experienced physiotherapists (> 10 years). One collected written information, and the other was in charge of physical performance tests.

Considering the fall definition provided by the World Health Organization, a fall is an event that results in a person coming to rest inadvertently on the ground or floor or other lower level (WHO, 2021). Participants were asked at baseline about the number of fall events before surgery (last year) with the question: “In the past 12 months, have you had any falls, including a slip or trip in which you inadvertently lost your balance and landed on the ground or lower level?”. Then, participants were provided with a calendar to record any possible fall event that occurred after undergoing surgery. We collected information on the number of fall events and requested that participants provide a narrative description. To avoid information loss, phone calls were made every 2 months as a reminder to fill in calendars during the 12 month follow-up period. In addition, participants were asked to attend face-to-face evaluation sessions scheduled at 6 and 12 months after surgery.

### Fall classification framework

A classification framework was applied considering published terminology and previous fall descriptors reported in patients undergoing TKR^20–22,29,30^ in older adults^33,34^ and mostly based on that previously developed for other lower limb prosthesis users^35^. Considering biomechanical theory, a fall occurs when the center of mass (CoM) does not fall within the base of support (BoS) or, in other words, when the so-called line of gravity—an imaginary line that crosses vertically the CoM—does not pass through the BoS. This produces an instability that, if not corrected through the mechanisms of postural control—such as foot, ankle or step movement strategies, or through a sufficient muscle strength that is capable of correcting such instability^36^^,^^37^ will precipitate a fall event. This instability occurs for two main reasons: due to a disturbance (e.g., force) that acts in the BoS itself and modifies its size/shape or displaces it beyond the center of gravity, in such a way that the line of gravity no longer passes through the BoS, or due to a disturbance or destabilization that acts above the BoS and displaces the CoM in such a way that the line of gravity no longer passes through the BoS (see Appendix 1). It is necessary to say that some falls are not biomechanical in nature, for instance, when they originate from physiological factors, such as dizziness and vertigo.

According to these principles, a three-level system was proposed. The first level (L1) was defined to classify the location of the destabilizing force with respect to the body so that a fall can result from a destabilization force in the CoM or the BoS. However, if the provided fall-description did not allow to classify the event within said categories, the fall was classified within the category 'Others', as afore argued. Accordingly, the L1 had three categories, L1 = [CoM; Bos; Others], considering whether the destabilizing force displaced the CoM beyond its BoS (L1[CoM] = 1), or the BoS beneath the CoM (L1[BoS] = 1)^31^. Those falls that could not be categorized as biomechanical in origin were classified as others, i.e. L1[Others] = 1.

The second level (L2) considered the source of destabilization. This level was classified depending on whether the disruptions were due to intrinsic factors, which are physiologic in nature (e.g. muscle weakness) or extrinsic factors, which are due to external/environmental factors, as the source that precipitated the destabilization (e.g., unstable ground). Once a fall was classified within a L1 category, was then classified within L2 categories, L2 = [Int; Ext; Others]. The L2[Others] category was defined for those falls due to physiological (non-biomechanical) factors (e.g. dizziness); it becomes necessary to clarify that, given their non-biomechanical nature, falls classified as L1[Others] = 1 were also classified as L2[Others] = 1.

The third level (L3) described the main fall-precipitating factor according to common and specific terminology on the topic and frequently repeated fall circumstances^20–22,29,30,33^. The established categories for the main precipitating factors were L3 = [Trip; Slip; Inadequate BoS; Others] in case of L1 = [BoS] and L2 = [Ext, Int] falls. In addition, in the event that L1 = [CoM] and L2 = [Ext], then L3 = [Push; Pull]. Finally, when L1 = [CoM] and L2 = [Int], then L3 = [Turning; Transferring; Standing; Climbing; Walking; Others]. The elaboration of these concepts, a detailed description of each level and category, as well as a diagram of the classification framework, are shown in Appendix 1.

### Measures

The characteristics of participants in terms of self-reported functionality, functional performance, knee function (strength) and pain were assessed. Specifically, self-reported functionality was measured using the Spanish version of the Oxford Knee Score, a 12-item questionnaire of different dimensions of knee pain and function, with items scoring from 0, the worse, to 4, the best function, to reach a maximum score of 48 points (ICC = 0.993)^38^. The timed up and go test, a timed test of general mobility and functional performance used to estimate fall risk and dynamic balance, in which the participant was instructed to get up from an arm-chair, walk for three meters, turn around a cone and come back to sit again (ICC = 0.87–0.99)^39^. The single-leg stand test, a timed test that was used to measure the participant stability on one limb, was delivered by measuring the time a participant could stand on surgical and contralateral limbs (ICC = 0.86–0.91)^40^. Knee extensor strength was measured with the participant sited on a Colson chair using a hand-held dynamometer (model 01165; Lafayette®), with the knee and hip flexed 90º, and the dynamometer located in the distal third of the tibia, secured with a belt around the limb and chair (ICC = 0.92–0.97)^41^. A visual analog scale was used to assess knee pain in the last week, considering 0, no pain, to 10, the worst possible pain^42^.

### Data analysis

A descriptive synthesis of the characteristics of participants was made with the software SPSS 22.0 (IMB®) licensed by Universitat de València using means, standard deviations, frequencies and contingency tables. Regarding falls, participants were classified as fallers (at least one fall) or nonfallers. Fall incidence with a confidence interval (CI) of 95%, along with the total number of falls, falls per patient and falls per faller, were estimated at baseline and at 6 and 12 months after surgery. Then, postoperative falls were classified by two researchers independently using the aforementioned proposed framework, first to determine the location of the destabilization force, then to determine the source of disruption and finally to determine the fall-precipitating factor. In addition, the causes of falls (e.g., trip, slip) and the activity during falls (e.g., walking, climbing) were described. Sample distribution and normality were tested with Shapiro–Wilk and Levene tests. Then, one-way analysis of variance based on a mixed model of repeated measures compared the characteristics of participants (independent variable = faller/nonfaller). The odds of relapse and its percentage probability were estimated (95% CI). The odds of falling when fallers used walking aids (yes/no) or exercised regularly (yes/no, we considered that a participant exercised regularly if he or she performed some type of physical activity at least three times per week for 30 min or longer).

### Ethical approval

The design adhered to the ethical recommendations set in Helsinki and successive updates and was approved by the ethics committees of the Hospital Universitari i Politècnic La Fe and Hospital Clínic i Universitari from Valencia (no. 2018/0621 and no. 2018/280).

### Informed consent

All participants signed a consent form.

## Results

The included sample was aged 71.4 (SD 6.4), of which 169 (66.5%) were women. There were 9 participants lost to follow-up at 6 months and 39 at 12 months. Common comorbidities were diabetes (53, 21%), arterial hypertension (159, 63%), high cholesterol (114, 45%), and some type of cancer (33, 13%). Figure 1 shows the flow of participants throughout the study.

### Characteristics of participants

Basal assessments suggested that fallers presented a lower capacity of maintaining balance on the surgical limb and lower knee extensor strength in such limbs than nonfallers (*p* < 0.05). Their self-reported function was also lower at 6 months after surgery (*p* < 0.05). In terms of pain, overall mobility (timed up and go), monopodal stability (single-leg balance) and contralateral knee strength, the data suggested no difference between fallers and nonfallers (*p* > 0.05). Measures of pain, self-reported functionality, balance and knee function are detailed in Table 1.

**Table 1:** Characteristics of participants over the study.

Characteristics	Overall	Fallers	Non Fallers	*p-*value^+^	*F*	Overall	Fallers	Non Fallers	*p-*value^+^	*F*	Overall	Fallers	Non Fallers	*p-*value^+^	*F*
n	253	102	151			244	32	212			214	50	164		
Age (years)	71.4 (6.0)	71.2 (7.5)	71.4 (6.7)	0.763	0.099	71.1 (6.5)	68.4 (7.4)	71.1 (6.6)	0.266	1.255	71.1 (6.2)	70.0 (6.2)	70.8 (6.2)	0.465	0.537
Sex (women, %)	177 (70%)	81 (45%)	96 (55%)	**0.029**		166 (68%)	24 (14%)	142 (86%)	0.521		150 (70%)	38 (25%)	112 (75%)	0.461	
BMI (kg / m^2^)	31.4 (5.8)	31.0 (5.2)	31.6 (6.2)	0.383	0.764	31.61 (6.8)	31.3 (5.8)	31.8 (7.4)	0.583	0.303	31.9 (6.3	32.1 (4.8)	31.7 (7.1)	0.807	0.060
Walking aid (y, %)	99 (40%)	48 (47%)	51 (53%)	**0.034**		64 (26%)	10 (15%)	54 (85%)	0.428		54 (25%)	12 (22%)	42 (78%)	0.604	
**Lifestyle**															
Regular exercise (y, %)	142 (56%)	60 (42%)	82 (58%)	0,505		194 (77%)	18 (9%)	176 (91%)	**0.030**		146 (68%)	16 (23%)	130 (77%)	0,523	
Residence (city, %)	187 (74%)	67 (36%)	120 (64%)	0,482		183 (75%)	20 (11%)	163 (89%)	0.920		161 (75%)	120 (75%)	41 (25%)	0,114	
**Measures**															
OKS (score 0–48)	38.6 (9.2)	40.7 (8.1)	39.5 (8.8)	0.177	1.753	25.9 (9.9)	31.3 (11.0)	25.4 (9.7)	**0.049**	3.824	22.3 (8.1)	23.9 (10.7)	21.7 (7.2)	0.335	0.944
TUG (s)	16.2 (6.3)	16.6 (7.4)	15.8 (6.7)	0.321	0.990	12.9 (4.5)	11.9 (2.9)	13 (4.7)	0.512	0.433	13.5 (6.8)	12.6 (6.1)	13.9 (7.1)	0.559	0.346
VAS pain (0–10)	5.7 (2.2)	5.9 (2.1)	5.6 (2.3)	0.428	0.572	2.8 (2.6)	3.6 (3.0)	2.7 (2.6)	0.389	0.749	2.6 (2.4)	2.8 (2.2)	2.7 (2.4)	0.732	0.118
OLB surgical knee (s)	7.3 (8.6)	5.1 (7.3)	8.4 (9.3)	**0.032**	4.658	9.4 (10.3)	6.8 (9.3)	9.7 (10.5)	**0.043**	4.218	11.3 (10.9)	9.4 (10.3)	11.4 (11.1)	**0.027**	5.321
OLB CL knee (s)	8.2 (9.8)	7.0 (9.8)	9.5 (9.9)	0.087	2.943	10.4 (10.2)	6.1 (9.5)	10.9 (10.3)	0.183	1.798	9.3 (11.4)	11.2 (11.8)	9.3 (11.4)	0.550	0.360
Strength surgical knee (kg)	16.9 (6.9)	15.8 (5.7)	17.6 (6.1)	**0.049**	3.952	21.6 (9.3)	21.2 (4.6)	21.6 (9.7)	0.912	0.012	22.2 (8.5)	19.3 (6.2)	23.1 (8.9)	**0.022**	5.417
Strength CL knee (kg)	18.9 (6.5)	18.1 (6.5)	19.5 (6.6)	0.165	1.942	22.1 (7.8)	21.5 (5.0)	22.1 (8.0)	0.816	0.054	23.6 (9.0)	21.1 (9.3)	24.4 (8.9)	0.201	1.668

Data are given as the mean (SD).

BMI, body mass index; OKS, oxford knee score; TUG, timed up and go; VAS, visual analogue scale of pain; OLB, one-leg stand balance test; CL, contralateral

+ *p*-value of F-test or Chi-square test for continuous or categorical variables, respectively. Significant values are in bold

Overall, 40% of participants used walking aids, and 56% exercised regularly before surgery; these participants were more likely to fall, with odds of OR = 1.7 (95% CI 1 to 2.9) and OR = 1.2 (95% CI 0.7 to 2), respectively. The use of walking aids decreased after surgery (approx. 25% of the sample); in this case, walking aid users were less likely to fall (OR = 0.26 to 0.12). In contrast, there was an increase in the practice of regular physical exercise, a factor that significantly decreased the odds of falling [OR_6m = 0.2 (0.1 to 0.5); OR_12m = 0.1 (0 to 0.2)].

### Incidence of falls

Overall, 102 participants were classified as fallers before TKR surgery; the fall incidence was 40.3% (95% CI 34.2% to 46.6%). This figure decreased to 13.1% (95% CI 9.2% to 18.0%) at 6 months after surgery; in the subsequent 6 months, the incidence increased to 23.4% (95% CI 17.8% to 29.6%). The probability of relapse was 71.2% (OR = 2.5, 95% CI 0.9 to 6.1). Information on falls is described in Table 2.

**Table 2:** Fall descriptors.

	Pre-surgery	Post-surgery (6 m)	Post-surgery (12 m)
Participants (n)	253	244	214
Fallers (n)	102	32	50
Fall prevalence (%, 95% CI)	40.3 (34.2–46.6)	13.1 (9.1–18.0)	23.36 (17.8–29.6)
Total falls (n)	277	44	108
Falls per patient	1.1	0.2	0.5
Falls per faller	2.7	1.4	2.2
Relapse probability (OR, 95% CI)	–	2.3 (1.2–4.5)	2.5 (0.9–6.1)
Relapse probability (%)	–	70.0%	71.1%

### Circumstances of falls

**Figure 2:**
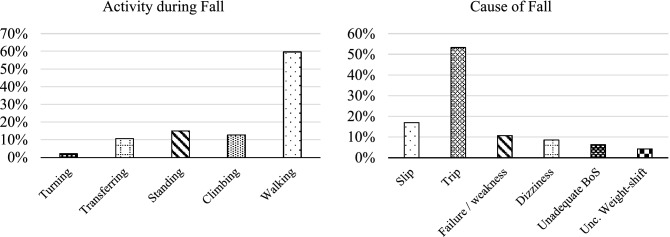
Activity performed during falls and main causes of falls represented as percentages.

Overall, falls occurred similarly at home (49%) and in other outdoor environments (51%). The majority of falls were in the forward direction (88%), occurred while walking (60%), and were mainly due to trips (53%) or slips (17%). Figure 2 summarizes the causes and activities performed during falls.

### Classification of falls

The BoS was the most frequent location where the destabilizing force precipitated a fall event (n = 102, 72%). Most of these were caused by extrinsic factors (n = 78, 76%) and trip patterns. CoM disruptions represented 19% of the reported falls, in this case, due to intrinsic factors. The falls not classified within BoS or CoM disruptions were physiological in nature and related to dizziness episodes. The details are shown in Table 3.

**Table 3:** Fall classification framework.

Location of destabilizing force	Source of destabilization	Precipitating factor
Base of Support (72%)	Intrinsic (24%)	Slip (12%)
		Trip (62%)
		Inadequate BoS (26%)
	Extrinsic (76%)	Slip (23%)
		Trip (73%)
		Uncorrected BoS (4%)
Center of Mass (19%)	Intrinsic (100%)	Slip (11%)
		Trip (11%)
		Uncorrected weight shift (22%)
		Muscle weakness (56%)
	Extrinsic (0%)	
Others (9%)	Physiological (100%)	Dizziness (100%)

Five of the 152 reported falls were not classified due to incomplete information.

### Suitability of the classification framework

The proposed framework was suitable since we classified 93% of falls. All the registered events could be classified within the first level according to our proposal. However, some of the classifications did not align with any reported event; for instance, no fall was classified as an extrinsic event that displaced the CoM beyond its BoS (ex. caused by a push, pull or collision). In addition, thirteen falls could not be classified because either the collected information was not complete or detailed enough, or patients could not remember some of the details of the event.

## Discussion

This study supported that falls are prevalent in individuals with severe knee osteoarthritis and that fall incidence decreases after undergoing TKR for at least one year, which was the monitoring period. Most fall events originated from disruptions in the BoS and were precipitated by extrinsic factors, generally trips during walking activities. The characteristics that significantly differentiated fallers and nonfallers were that fallers presented less monopodal stability and decreased strength in the surgical limb before and after surgery, as well as a more limited self-reported functionality after surgery. Further studies are needed to support these results, but our findings may help to develop clinical assessment methodologies and treatment strategies to reduce fall incidence.

The rate of fallers was within the range reported by previous studies on the topic^20–22,29,43^. Our results suggested that approximately 40% of individuals with severe knee osteoarthritis fell at least once in the year before surgery, which is consistent with previous data (23% to 63%)^8^. The registered fall incidence was 13% and 23% at 6 and 12 months after surgery, also within the range of previous reports (13% to 42%)^8,11^. The differences across studies may be related to multiple factors, including methods for data collection, patient sources, diverse time points for assessment or even the inclusion of fall prevention strategies in the recovery processes.

Most studies agreed that fall events were mainly due to trips and slips, and these occurred during walking^21,29^. Falls occurred similarly in indoor and outdoor environments, which was in contrast with the study of Chan et al.^29^ who reported an approximate 65% of falls at home, or Tsonga et al.^21^ who suggested right the opposite (i.e. 65% outdoors).

No previous study with patients undergoing TKR classified falls into a three-level system. However, Swinkels et al.^30^ suggested that preoperative falls were similarly distributed between those caused by intrinsic or extrinsic factors and that postoperative falls were mainly due to extrinsic factors, while Kim et al. proposed a similar classification for other lower limb prosthesis users^31^. Our study classified postoperative falls, and the results were in agreement with such findings. Additionally, it was revealed that most of the falls originated from forces that acted on the BoS, which helped to expand current knowledge but also to determine suitable strategies to reduce fall risk. On the other hand, the second level of characterization may help to identify recurrent fallers. Indeed, while the majority of the available balance assessment tools rely on intrinsic performances^31,44^, the findings suggested that extrinsic factors precipitated most destabilizations. Therefore, first, it seems necessary to develop tests that are capable of detecting the ability to respond to external disturbances, and second, to estimate the risk of falling or to detect potential fallers, it would be convenient to complement the usual tests that mostly evaluate the intrinsic capacities with measurements that are capable of estimating the response to external disturbances.

The association of some intrinsic factors with falls in older adults has been reported in the literature, including history of falls, advanced age, gender or muscle weakness, among others^32,45^. In addition, poorer operated knee proprioception and sensory orientation were also identified as potential contributors^29^. However, the findings across studies remain controversial in patients undergoing TKR. For instance, we agree with Chan et al.^29^ that knee instability is a factor that can help to identify fallers, but such a study did not find a significant association with a history of falls or knee strength. By contrast, Tsonga et al.^21^ found that a history of falls was a predictor of future fall events, which seems consistent with the relapse probability suggested in our study (over 70%). On the other hand, we found that surgical limb strength was a factor that differed significantly between fallers and nonfallers.

The contribution of knee pain to falls is also uncertain. Some studies suggested that a greater intensity of pain is associated with falls^29,46^, but others (including ours) did not find differences in knee pain among fallers and nonfallers^30^. A similar statement can be made with regard to physical function. It seems well established that as overall functional capacity improves after TKR, the incidence of falls decreases; however, previous association and regression analyses that compared fallers and no fallers did not find significant differences in these terms^20–22,29,30^. Overall, our results support this view, but our fallers presented a significantly greater self-reported functionality (only at 6 months postsurgery). Many studies have pointed out that inactivity can lead to less physical performance, increasing the risk of falls, while physically active individuals are more likely to engage in risky activities. Although this would justify the above, it would not explain how the registered increase in the practice of regular physical exercise after surgery significantly decreased the odds of falling. The results of this and other studies continue to suggest that the association between functional capacity, physical activity and falls is complex and is probably influenced by a combination of bio-psycho-social factors that require further study for a complete understanding^47^.

It is necessary to consider some limitations. A small number of patients, even when there was no obvious memory dysfunction, may not have accurately recollected the details of their falls, especially before surgery, when our data were based on retrospective information. This may have influenced the estimated fall incidence. The results can be generalized to only participants with similar characteristics, but future studies are warranted to support and expand our findings (for instance, including patients with bilateral or secondary TKR). We characterized patients according to their physical status but not according to other comorbidities, medications, social, economic or psychological and cognitive status, factors that may have some impact on our results^47^. The use of sensor-based technology, such as inertial systems, could have helped to accurately measure changes shape and size of the BoS, or even accurately quantify CoM changes (e.g. displacements, swayed areas and velocities); therefore, the use of these devices could provide additional information to complete the proposed framework, and are recommended for future investigations. Further research is needed to assess the suitability of the proposed classification framework.

## Conclusion

This study supports that falls are prevalent in patients with severe knee osteoarthritis before undergoing total knee replacement. Symptoms and functional performance improve after surgery, and fall incidence is reduced. Most fall events originate from disruptions in the BoS and are precipitated by extrinsic factors, generally trips during walking activities. In addition, fallers present less monopodal stability and decreased strength in the surgical limb than nonfallers, as well as a more limited self-reported functionality after surgery. Further studies are needed to support these results, but this work may help to develop clinical assessment methodologies and treatment strategies to reduce the reported incidence of falls.

## Supplementary Information

Supplementary Information 1.

Supplementary Information 2.

Supplementary Information 3.

## Data Availability

The datasets used and/or analyzed during the current study available from the corresponding author on reasonable request.

## Abbreviations

TKR: Total knee replacement
CoM: Center of mass
BoS: Base of support
