# Publisher Correction: Fall classification, incidence and circumstances in patients undergoing total knee replacement

**DOI:** 10.1038/s41598-022-26371-z

**Published:** 2022-12-16

**Authors:** José-María Blasco, José Pérez-Maletzki, Beatriz Díaz-Díaz, Antonio Silvestre-Muñoz, Ignacio Martínez-Garrido, Sergio Roig-Casasús

**Affiliations:** 1 Group in Physiotherapy of the Ageing Process: Social and Healthcare Strategies, Departament de Fisioteràpia, Universitat de Valéncia (Spain), Calle Gascó Oliag nº5, 46010 Valencia, Valencia Spain; 2 IRIMED Joint Research Unit, IIS La Fe - UV, Valencia, Spain; 3 Hospital Clínic i Universitari de València, Valencia, Spain; 4 Hospital Universitari i Politècnic La Fe, Valencia, Spain

Correction to: *Scientific Reports* 10.1038/s41598-022-23258-x, published online 18 November 2022

The original version of this Article erroneously included the Acknowledgements. It has consequently been removed.

The original Article has been corrected.

