# Supplementary material for: Fall classification, incidence and circumstances in patients undergoing total knee replacement
Source: Sci Rep. 2022 Nov 18;12:19839. doi: 10.1038/s41598-022-23258-x (PMC9674575; doi:10.1038/s41598-022-23258-x)
Supplement: Supplementary file 1 — Supplementary Information 1. [file 41598_2022_23258_MOESM1_ESM.docx]

**Appendix 1**

**Fall classification proposal. Rationale and definitions**

A fall is defined as an event which results in a person coming to rest inadvertently on the ground or floor or other lower level (WHO, 2021).

If analyzed in biomechanical terms, and although there may be exceptions, a fall occurs when the center of gravity does not fall within the base of support or, in other words, when the so-called line of gravity, that is, the line that crosses vertically the center of gravity, does not pass through the base of support. This produces an instability that if not corrected through the mechanisms of postural control, (such as foot, ankle or step movement strategies, or through a sufficient muscle strength that is capable of correcting such instability),^1,2^ will precipitate a fall event.

Said instability use to occur for two main reasons: first, due to a disturbance (e.g. force) that acts in the base of support itself and modifies its size/shape or displaces it beyond the center of gravity, in such a way that the line of gravity no longer falls within the base of support (figure 1, a). Second, due to a disturbance or destabilization that acts above the base of support and displaces the center of gravity in such a way that the line of gravity no longer falls within the base of support (figure 1, b).

It is necessary to say that some falls are not biomechanical in nature, for instance when they originate from dizziness, vertigo and etcetera, as is further detailed.


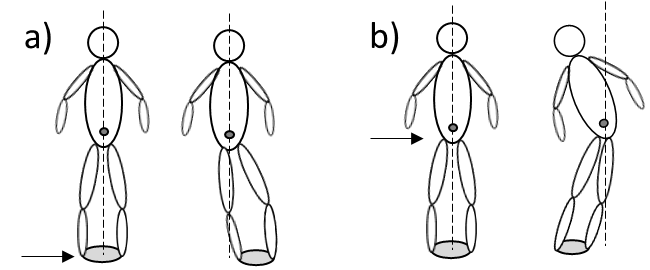


Figure A1.

Figure A2 shows the classification framework, in which a fall event can be classified, firstly, into those occurred by a disturbance or disruption in the base of support, in the center of mass or some other pace, secondly, according to the cause, which could be intrinsic or extrinsic to the body, and finally, according to the precipitating factor.


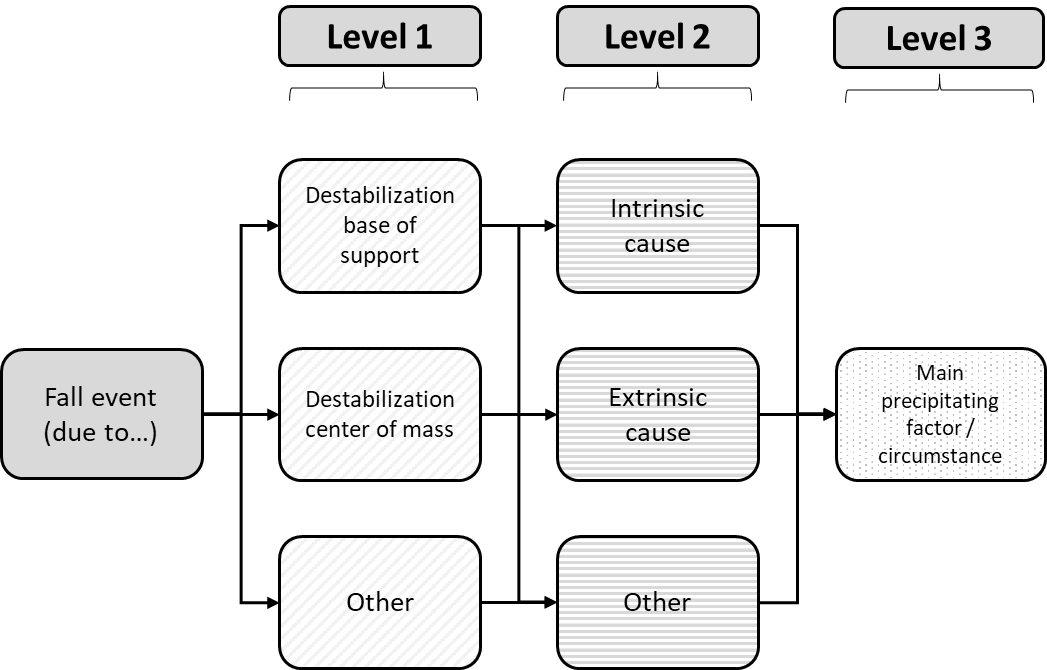


Figure A2

In the following lines this classification is further elaborated. Most of these definitions have been extracted or adapted from literature.^3–8^ Falls can be due to:

- **L1 [BoS] = Disturbance, disruption or perturbation in the base of support:** Any disturbance (e.g. force) that produces a displacement or modification of the base of support. It can be assumed that such the displacement/modification is important enough for the line of gravity to fall outside the base of support, producing an instability that precipitates the fall event.
  - **L2 [Ext]= Extrinsic/external cause:** The disturbance is caused by a factor external to the body, by catching the foot or leg on uneven surface, obstacle, step, or terrain transition – for instance, a stone on the road (**L3[Trip]**=main precipitating factor is a trip) –, or by inadequate friction between the foot and the ground, –for instance in a slippery floor (**L3[Slip]**=slip) –, or by a surface that does not allow full and/or flat support of the foot – meaning that the contact surface is small or uneven (**L3[Inadequate BoS]**= inadequate base of support) – or by any other external event (**L3[Others]**)
  - **L2 [Int]= Intrinsic / internal cause:** The foot path is modified in a stable and even surface, a technical aid, or the subject's own body,^3^ and this precipitates a fall. Therefore, the disturbance in the base of support is caused by a factor internal to the body such as a failure that produces a stumble (**L3[Trip]**=Trip), or a slip (**L3[Slip]**=slip) on the ground, or when the foot is not correctly placed in the ground, for instance when climbing a step (**L3[Inadequate BoS]**=inadequate support base)
- **L1 [CoM] = Disturbance, disruption or perturbation in the center of mass:** Any disturbance that produces a displacement or modification of the center of mass and, consequently, the subject falls. In principle, it can be assumed that such a modification/displacement is important enough for the line of gravity to fall outside the support base, producing an instability that precipitates the fall event.
  - **L2 [Ext]= Extrinsic / external cause:** possible external causes are an external force that is directly applied on the center of mass by pushing it –for instance, if another person’s push the body or if there is a collision with some other person or some object (**L3[Push]**) –, or by pulling from it (**L3[Pull]**).
  - **L2 [Int]= Intrinsic / internal cause:** possible internal causes occur when performing activities such as turning, reaching or transferring, and there is self-induced shifting of body weight beyond the base-of-support: **L3=[Turning; Transferring; Standing; Climbing; Walking; Others]**
- **L1 [CoM] = Other type of falls,** not biomechanical in nature (extracted from Kim et al., 2019):
  - **L2 [Ext]= Extrinsic**: fall initiated by a support structure moving unexpectedly, (e.g. chair moves unexpectedly during sit-to-stand).
  - **L2 [Int]= Intrinsic:** examples are falls due to prosthesis factors (**L3[Mal-functioning; Mal-alignment; Others]**), or those due to physiological factors (**L3[muscle weakness; dizziness; others]**).

**References**

1. Horak, F. B. Postural orientation and equilibrium: what do we need to know about neural control of balance to prevent falls? *Age and Ageing* **35**, ii7–ii11 (2006).

2. Sibley, K. M., Beauchamp, M. K., Van Ooteghem, K., Straus, S. E. & Jaglal, S. B. Using the systems framework for postural control to analyze the components of balance evaluated in standardized balance measures: a scoping review. *Archives of physical medicine and rehabilitation* **96**, 122-132.e29 (2015).

3. Kim, J., Major, M. J., Hafner, B. & Sawers, A. Frequency and Circumstances of Falls Reported by Ambulatory Unilateral Lower Limb Prosthesis Users: A Secondary Analysis. *PM and R* **11**, 344–353 (2019).

4. Reed, A. T. *et al.* Falls in the Elderly: Reliability of a Classification System. *Journal of the American Geriatrics Society* **39**, 197–202 (1991).

5. Topper, A. K., Maki, B. E. & Holliday, P. J. Are activity-based assessments of balance and gait in the elderly predictive of risk of falling and/or type of fall? *Journal of the American Geriatrics Society* **41**, 479–487 (1993).

6. Robinovitch, S. N. *et al.* Video capture of the circumstances of falls in elderly people residing in long-term care: an observational study. *Lancet (London, England)* **381**, 47 (2013).

7. Tsonga, T. *et al.* Reduction of Falls and Factors Affecting Falls a Year After Total Knee Arthroplasty in Elderly Patients with Severe Knee Osteoarthritis. *The Open Orthopaedics Journal* **10**, 522–531 (2016).

8. Chan, A. C. M., Jehu, D. A. & Pang, M. Y. C. Falls after total knee arthroplasty: Frequency, circumstances, and associated factors-a prospective cohort study. *Physical Therapy* **98**, 767–778 (2018).
